# Long-term survival for ICU patients with acute kidney injury

**DOI:** 10.1186/cc10983

**Published:** 2012-03-20

**Authors:** D Scott, F Cismondi, J Lee, T Mandelbaum, LA Celi, RG Mark, D Talmor

**Affiliations:** 1 MIT, Cambridge, MA, USA; 2 Beth Israel Deaconess Medical Center, Boston, MA, USA; 3 Sheba Medical Center, Tel Hashomer, Israel

## Introduction

A recently published study [1] validated the criteria used in the Acute Kidney Injury Network (AKIN) definitions [2] of the three stages of acute kidney injury (AKI) using in-hospital mortality. In the present study, we validate the clinical applicability of the AKIN classifications through long-term survival analysis of AKI patients.

## Methods

From over 17,000 adult ICU patients in the MIMIC II database [3,4] (V2.5), we excluded patients having end-stage renal disease and those with insufficient data and determined AKI stages for each patient. Multivariate Cox regression was used to determine hazard ratios (HRs) for 2-year survival, controlling for: age, sex, nonrenal Sequential Organ Failure Assessment (SOFA) score and selected co-morbidities.

## Results

Among the final cohort of 14,525 patients, 43% had no AKI and 39%, 14% and 4% developed AKI 1, 2 and 3 respectively. The results of the regression analysis show that AKI 1 (HR 1.12, *P *< 0.05), AKI 2 (HR 1.10, *P *= 0.05) and AKI 3 (HR 1.64, *P *< 0.001) were significantly associated with increased 2-year mortality. In addition, age (HR 1.04, *P *< 0.001), gender (M) (HR 0.93, *P *< 0.05), nonrenal SOFA score (HR 1.05, *P *< 0.001) and all co-morbidities were significant predictors. Adjusted and unadjusted Kaplan-Meier curves for patients with AKI 3 are remarkably different from each other, suggesting that in these most severely ill patients AKI is only one aspect of their illness.

## Conclusion

AKI stages 1, 2 and 3 are significant indicators of 2-year mortality. The difference between AKI 1 and 2 is smaller than that between AKI 2 and 3 and it may be prudent to re-examine the criteria used to define AKI to provide better separation among the three classes.
